# Hearing Aids Reshape Neural Processing of Emotional Speech Without Improving Emotion Perception

**DOI:** 10.1177/23312165261465515

**Published:** 2026-07-02

**Authors:** Carmen Dang, Gurjit Singh, Frank A. Russo

**Affiliations:** 1 Department of Psychology, 443337 Toronto Metropolitan University, Toronto, ON, Canada; 2 Department of Speech-Language Pathology, University of Toronto, Toronto, ON, Canada; 3 Sonova Canada, Kitchener, ON, Canada

**Keywords:** speech emotion perception, functional near-infrared spectroscopy, hearing aids, hearing loss, aging

## Abstract

Hearing aids have improved speech intelligibility but not speech emotion perception in older adults with hearing loss. Hearing loss has also been linked to altered brain responses to emotional non-speech sounds, potentially contributing to the speech emotion perception deficits even under aided listening. While fMRI requires hearing aid removal due to metal incompatibility, fNIRS is silent and hearing-aid compatible, creating a novel opportunity to identify neural processes during aided listening. We leveraged fNIRS to examine how hearing aids affect brain function in experienced hearing-aid users during speech emotion perception. Older adults (17 normal hearing, 14 hearing-aid users) judged vocal-emotion changes in speech while fNIRS recorded hemodynamic activity. Hearing-aid users completed the task under aided and unaided listening. Behaviorally, hearing aids did not improve speech emotion perception, replicating prior research. In cortical responses, individuals with hearing loss showed increased planum temporale activity, regardless of hearing-aid use. Unaided listening showed additional frontal recruitment, including increased medial superior frontal gyrus activity and stronger functional connectivity between inferior frontal and superior temporal gyri. Conversely, aided listening increased inferior parietal lobe activity, but reduced connectivity between inferior frontal gyrus and inferior parietal lobe. Together, findings indicate that hearing aids partially modify cortical processing of emotional speech. They reduced reliance on frontal compensatory control systems and increased engagement of a higher-order parietal region, consistent with partial restoration of bottom-up auditory information during aided listening. Persistent abnormalities in early auditory processing and incomplete sensorimotor integration may explain why hearing aids fail to normalize speech emotion perception.

## Introduction

Human communication extends beyond the literal meaning of words; it is enriched by vocal emotional cues such as pitch, intensity and prosody. However, changes in our hearing may impact our ability to perceive emotions in speech (Picou et al., 2018). While hearing loss at any age may impair speech emotion perception (e.g., Rachman et al., 2025), the present paper focuses on hearing loss in older adults. Hearing loss impairs perception of sound frequencies, primarily high-frequency sounds, and has been linked to speech emotion perception deficits. For instance, older adults with hearing loss were worse at perceiving emotions in speech than those with normal hearing (Singh et al., 2019).

Although vocal pitch, an acoustic cue to vocal emotion (Banse & Scherer, 1996; Major & Chatterjee, 2026), is conveyed in the lower frequencies, recent research has linked high-frequency loss to speech emotion perception deficits. In an adult sample (ages 19 to 72), mild and moderate hearing loss only in the higher frequencies (3, 4, 6 and 8 kHz) predicted worse speech emotion perception compared to normal hearing (Chatterjee et al., 2026). This may be explained by several factors, one of them being that in addition to pitch, spectral balance is an acoustic cue important for vocal emotion perception. For instance, acoustic analyses have found that emotional speech differs from neutral speech in the balance of energy between low (1-5 kHz) and high (5-8 kHz) frequencies (Guzman et al., 2013). Similarly, while controlling for other acoustic cues like mean pitch, acoustic analyses found that the spectral centroid predicted participant ratings of arousal and valence of vocal emotion (Sauter et al., 2010). Given that the relative balance of low- and high-frequency sounds is a cue to vocal emotion, restoring audibility across frequencies in those with hearing loss may improve speech emotion perception.

Hearing aids are one of the primary treatments for hearing loss, improving audibility through frequency-specific amplification tailored to an individual’s hearing profile. However, many hearing difficulties persist despite amplification. While wearing hearing aids improved word perception compared to unaided listening, hearing aids did not improve speech emotion perception in older adults with hearing loss (Goy et al., 2018).

### Altered Neural Processing in Adults With Hearing Loss

From functional magnetic imaging (fMRI) research, even mild hearing loss has been linked to changes in neural activation when listening to affective sounds. Specifically, adults with sensorineural hearing loss showed increased engagement of parietal areas and reduced engagement of temporal, frontal and occipital areas compared to normal-hearing controls (Husain et al., 2014). These neuroplastic changes may also explain in part the deficits in speech emotion perception, even when using hearing aids. However, it is unclear whether these fMRI findings generalize to emotional speech or aided listening.

In addition to localized activations, fMRI research has also found differences in functional connectivity—the coactivation (i.e., temporal correlation) of distinct brain regions (Friston, 1994)—as a function of hearing acuity. Compared to normal-hearing, adults with profound bilateral hearing loss (>90 dB HL in each ear) showed greater task-based connectivity between an auditory area (i.e., superior temporal gyrus) and areas within an attention network (i.e., frontoparietal network). Moreover, greater connectivity between an auditory and attention area was linked to faster response times for the hearing loss group only (Song et al., 2023). These results suggest that hearing loss is linked to compensatory neuroplasticity of top-down control processes. However, the participants completed a visual task during neuroimaging so it is unclear whether these findings generalize to an auditory task. Furthermore, participants did not wear hearing aids during neuroimaging, leaving open the question of how hearing-aid use may influence brain networks.

### Functional Near Infrared Spectroscopy

In auditory research, studying the brains of hearing-aid users remains challenging because of important limitations associated with fMRI (Peelle, 2014). First, hearing aids typically contain ferromagnetic components and therefore must be removed during scanning, precluding the study of aided listening under naturalistic conditions. Second, scanner noise can mask target stimuli, modulate neural activity (Pellegrino et al., 2022; Tomasi et al., 2005) and disproportionately affect listeners with hearing loss, particularly those with reduced dynamic range or hyperacusis, who may experience sounds as uncomfortably loud or even painful (Husain et al., 2022). In contrast, functional near-infrared spectroscopy (fNIRS) is a noninvasive optical neuroimaging technique that is silent and compatible with hearing aids, making it an increasingly popular and promising tool in auditory cognitive neuroscience (Harrison & Hartley, 2019; Pinti et al., 2020; Shatzer & Russo, 2023).

Functional NIRS indirectly measures neuronal activity by emitting near-infrared light onto the scalp through to the cortex and detecting the backscattered light to capture changes in hemoglobin concentration. Hemoglobin is a protein in red blood cells that can be oxygenated (i.e., oxyhemoglobin; HbO) or deoxygenated (i.e., deoxyhemoglobin; HbR). An increase in HbO relative to a baseline is presumed to indicate neuronal activity because the brain metabolizes oxygen as energy when it is active. Across multiple cognitive tasks, NIRS signals are often highly correlated with fMRI signals, making it an appropriate alternative to fMRI (Cui et al., 2011).

Although the spatial resolution of fNIRS is inferior to that of fMRI and its imaging depth is limited to approximately 2–3 cm, many cortical regions implicated in speech emotion perception are in the superficial gyri (primarily Layers I to III), making them accessible to fNIRS (e.g., Alba-Ferrara et al., 2011; Beaucousin et al., 2011). These cortical areas encompass key components of the speech processing network and are now considered highly relevant for emotion perception. Unlike earlier theories that mapped discrete emotions onto discrete brain regions (Ekman, 1999), constructionist models propose that emotional experiences emerge from dynamic interactions among distributed cortical and subcortical systems involved in prediction, allostasis, and interoception (Barrett, 2016). Prior work has successfully measured localized activity using fNIRS across frontal, temporal and parietal areas, primarily in normal-hearing young adults. For example, relative to silence, the inferior frontal gyrus engagement (IFG) showed increased engagement during prosodic speech perception (Zhang et al., 2018). During complex social communication like cooperative gameplay, an fNIRS study found greater inter-brain synchrony between players in the medial prefrontal cortex (MPFC), dorsolateral prefrontal cortex (DLPFC) and superior frontal gyrus (SFG; Liu et al., 2016). Within temporal areas, fNIRS captured increased superior temporal gyrus (STG) and planum temporale (PT) activity while listening to emotional versus neutral prosody (Zhang et al., 2018). Lastly, fNIRS has identified elevated inferior parietal lobe (IPL) activity in a social imitation study (Oliver et al., 2018). Recently, fNIRS has been used to understand contributions of hearing aids to reducing listening effort in hearing-aid users. It has detected differences in prefrontal cortical activation during aided versus unaided listening (Vaisberg et al., 2024) and between standard and AI-based hearing aid noise management programs (Vaisberg et al., 2025).

Functional NIRS can also be used to study brain networks through functional connectivity (see Tak and Ye (2014) for a review). Brain networks measurable by fNIRS are defined by pairs of superficial cortical regions (i.e., node pairs). Relevant to the current study are node pairs from the dual-stream model of auditory and speech processing (e.g., IFG-IPL, STG-IFG, STG-IPL). This model proposes two specialized auditory processing pathways: a ventral stream for sound identification and a dorsal stream for processing complex sounds through sensorimotor simulation. The dual-stream model is prominent in speech perception and recently extended to prosody perception, making these node pairs particularly relevant for studying speech emotion perception (Rauschecker & Scott, 2009; Sammler et al., 2015). In particular, the IFG–IPL node pair is of interest because it has also been proposed as the default route in the dual-systems model of emotion perception (Preston & De Waal, 2002; Prochazkova & Kret, 2017; Shamay-Tsoory, 2011; Yu & Chou, 2018). Lastly, since aforementioned fMRI studies have linked hearing loss to differences in functional connectivity within attention networks like the frontoparietal network (FPN; Song et al., 2023), the IPL–DLPFC node pair is also of interest.

### Current Project

Behavioural studies on speech emotion perception in older adults suggest that hearing loss negatively impacts speech emotion perception and hearing aids do not provide significant benefit (see Picou et al., 2018 for a review). However, these studies often use a forced-choice response style task that may lack sensitivity to detect benefits of hearing-aid use. Crucially, there is a lack of neuroimaging studies on hearing-aid users, especially in the context of speech emotion perception. This gap may stem from limitations of fMRI scanner noise and incompatibility with metal devices, but fNIRS is growing increasingly popular in auditory research and has been successful in contributing to research on social cognition as well as speech understanding with hearing aids. Accordingly, the current study utilizes fNIRS to identify how hearing loss and hearing aids alter the neural processing of emotional speech, providing a neurocognitive account of speech emotion perception in older adult hearing-aid users.

Aligned with the current literature, we hypothesized that normal-hearing older adults would have better performance on our speech emotion perception task than those with hearing loss, regardless of hearing-aid use. Prior research has not found differences in performance on speech emotion perception tasks between aided and unaided listening using forced-choice paradigms, so here we tested the hypothesis that these conditions differ in our present task. Since our emotion-change task was designed to better capture subtle differences in speech emotion perception than the forced-choice paradigms used in the literature, it is plausible that aided listening would outperform unaided listening (after Schmidt et al., 2016). For the neural data, we hypothesized differences in HbO in at least one of the a priori regions of interest (ROI) between older adults with normal hearing, hearing loss aided and hearing loss unaided. Group differences in auditory regions were plausible given the inherent differences in the auditory signal due to hearing loss and hearing aids. It was also plausible to expect group differences in top-down control areas in response to a degraded auditory input. We also explored group differences in task-based functional connectivity for the a priori node pairs of interest.

## Materials and Methods

### Participants

Thirty-two participants were recruited from the Toronto Metropolitan University Participant Information Database. One participant was removed due to a procedural error wherein the participant did not adjust the volume to their most comfortable listening level, resulting in a final sample of 31 participants. There were 17 with normal hearing (10 Female, *M*_*age*_ = 69.70, *SD*_*age*_ = 4.09) and 14 bilateral hearing-aid users (8 Female, *M*_*age*_ = 74.30, *SD*_*age*_ = 7.36). Data for one participant’s aided listening condition was removed because they lost one of their hearing aids the week of their session. The Research Ethics Board at Toronto Metropolitan University (protocol number 2020-221) approved this study.

Using a Qualtrics online questionnaire (Qualtrics, Provo, UT), all participants self-reported to be in at least average health, have normal or corrected-to-normal vision, be native English speakers (i.e., learned English in Canada by age 5), and have not suffered from any head trauma (e.g., concussion) or neurological/psychological disorders (e.g., stroke, Parkinsons, depression). Participants also had no alexithymia, as assessed by Toronto Alexithymia Scale, and had none-to-low tinnitus handicap based on the Tinnitus Handicap Inventory. As assessed by the MoCA Audiovisual, all participants had normal cognition.

**Figure 1.**
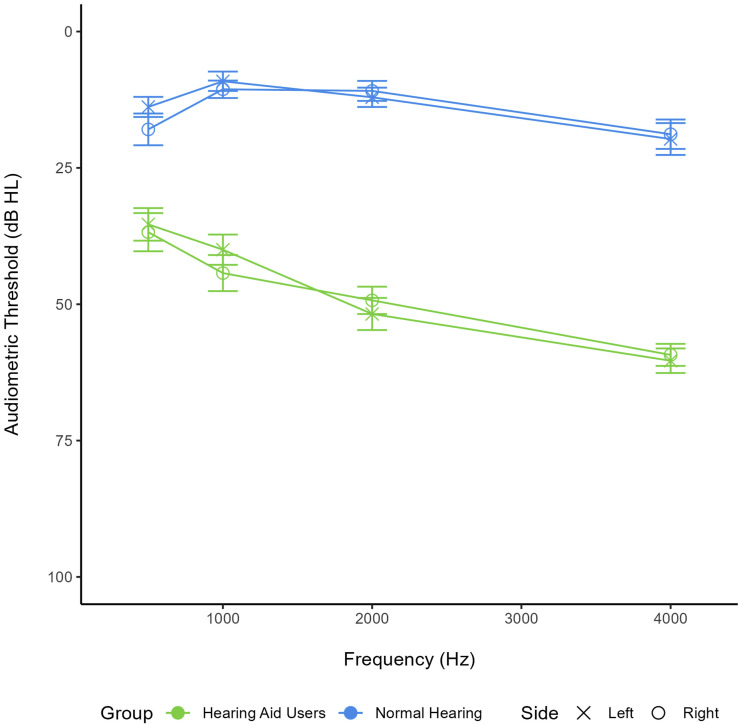
Group means of audiometric pure-tone thresholds. Error bars represent standard error

Hearing acuity was assessed by pure-tone audiometry with a calibrated clinical audiometer (GSI 61, Grason-Stadler, United States). Pure-tone thresholds were tested for each ear across eight frequencies (i.e., 250, 500, 1000, 2000, 3000, 4000, 6000 and 8000 Hz) and measured in decibels hearing level (dB HL). A pure tone average (PTA) was calculated across the following frequencies: 500, 1000, 2000 and 4000 Hz. All participants had symmetrical hearing defined as ≤ 15 dB HL difference in the PTA between ears. Normal-hearing participants had PTA of ≤ 25 dB HL across both ears and self-reported having never worn hearing aids. Hearing loss participants had an unaided PTA of ≥ 34 dB HL across both ears. They were also experienced (at least 1 year), regular (at least 4-8 hours a day) and satisfied (according to the Satisfaction with Amplification in Daily Life scale) users of bilateral hearing aids. Please see Table 1 for sample descriptive statistics, Figure 1 for audiometric thresholds, and Supplemental Material 4 for hearing-aid style, brand and model. Participants’ self-reported skin tone and hair type are presented in Supplemental Material 1 (Yücel et al., 2024).

**Table 1.** Sample Descriptive Statistics

Variables	Normal hearing (*N* = 17)				Hearing-aid users (*N* = 14)			
	M	SD	Min	Max	M	SD	Min	Max
Age (years)	69.70	4.09	64	80	74.30	7.36	62	85
4F PTA (dB HL)	14.10	5.69	5	23.80	47.10	7.47	34.40	62.50
MoCA	27.80	1.35	25	30	27.20	1.42	25	30
TAS	40.40	6.66	29	55	42.9	6.64	30	51
THI	0	0	0	0	14	15.6	2	36
Mean SADL	-	-	-	-	5.41	0.66	4	6.44
Age onset HL	-	-	-	-	64.07	8.84	47	75
Hearing-aid experience (years)	-	-	-	-	6.61	4.51	2	20

*Note.* Only participants who self-reported experiencing tinnitus completed the THI (*n* = 2 normal-hearing participants and *n* = 4 hearing-aid users). HL = hearing loss, MoCA = Montreal Cognitive Assessment, TAS = Toronto Alexithymia Scale, THI = Tinnitus Handicap Index, SADL = Satisfaction with Amplification in Daily Living, 4F PTA = four-frequency pure tone average calculated across 500, 1000, 2000 and 4000 Hz across both ears.

### Study Design

The independent variables were hearing acuity (between-groups) and hearing-aid use (within-groups). The between-groups comparisons were: 1) normal hearing vs. hearing loss aided and 2) normal hearing vs. hearing loss unaided whereas the within-groups comparison was between hearing loss aided vs. hearing loss unaided. The dependent variables were performance on our speech emotion perception task, changes in HbO from baseline, and task-based functional connectivity measured by fNIRS.

Task order (pure-tone audiometry vs. speech emotion perception task) was randomly assigned to each participant. In the speech emotion perception task, emotion pairs (i.e., happy-sad, angry-calm, sad-happy, calm-angry) were presented in blocks, with block order randomly assigned to each participant per session. To limit the number of false positive responses, emotion pairs were chosen based on their maximal distance on the dimensional model of emotion (i.e., valence and arousal, Russell, 1980). Hearing-aid users wore their own hearing aids and whether their hearing aids were worn in the first or second session was also randomized.

### Procedure

Participants attended a 90-minute in-lab session to complete pure-tone audiometry and the speech emotion perception task during an fNIRS recording. Hearing-aid users returned for a second in-lab session for 60 minutes on a separate day to complete the computer task again since they completed the task twice, once with and once without their own hearing aids. Participants set their hearing aid to their preferred settings.

### Stimuli

The full Ryerson Audio-Visual Database of Emotional Speech and Song (RAVDESS) includes audio, visual and audio-visual recordings of 24 North American professional actors (12 female) singing and speaking neutral statements (*Dogs are sitting by the door”* and *“Kids are talking by the door”*) in eight emotions (neutral, calm, happy, sad, angry, fearful, surprise and disgust) at two levels of intended intensity (normal and strong) and a neutral expression. Each clip is approximately 3 seconds in length and was recorded twice. In the original validation study, 247 undergraduate students from Toronto, Canada rated the clips on emotional validity, intensity, and genuineness. The sample rated the expressions as reflecting the intended emotional category with moderate-to-substantial levels of interrater reliability and high test-retest reliability (Livingstone & Russo, 2018).

A subset of 160 stimuli was selected from the RAVDESS for the speech emotion perception task. First, the database was filtered for the speech, auditory-only recordings expressing the emotions angry, sad, happy and calm. Next, it was further filtered to contain stimuli that had a median genuineness rating of at least 4 (on a Likert scale of 1 - not genuine to 5 - very genuine) and proportion correct between 60 to 90% from the original validation study (Livingstone & Russo, 2018). Actors were then ranked based on the number of their recordings that met these criteria, from highest to lowest. All recordings from the top five actors were used for the speech emotion perception task (a total of 32 files per actor: 4 emotions by 2 statements by 2 intensities by 2 repetitions). Recordings from the top four actors were used in experimental trials and those from the fifth actor were used in practice trials. All stimuli were equated on their average amplitude level through root mean square (RMS) normalization in Audacity (Muse Group, Cyprus) with a threshold level of -24.0 dB below digital full scale. See Supplemental Material 5 for stimulus set filenames.

### Behavioural Measure

#### Set-Up

Visual stimuli (i.e., instructions, fixation cross and text indicating the emotion pair per trial) were presented on a 1080p monitor approximately 65 cm away from the participant and auditory stimuli played from a loudspeaker positioned 85 cm in front of the participant and 135 cm above the ground. Prior to the task, participants listened to a sample of the task stimuli and adjusted the volume to their most comfortable listening level such that they heard each emotion clearly without the audio being uncomfortably loud (see Supplemental Material 2 for the sound level of each participant per group). The sample stimulus set comprised eight recordings: two per emotion (one normal- and one strong-intensity), with each actor sampled at least once and an equal number of recordings for each RAVDESS statement.

#### Speech Emotion Perception Task

**Figure 2.**
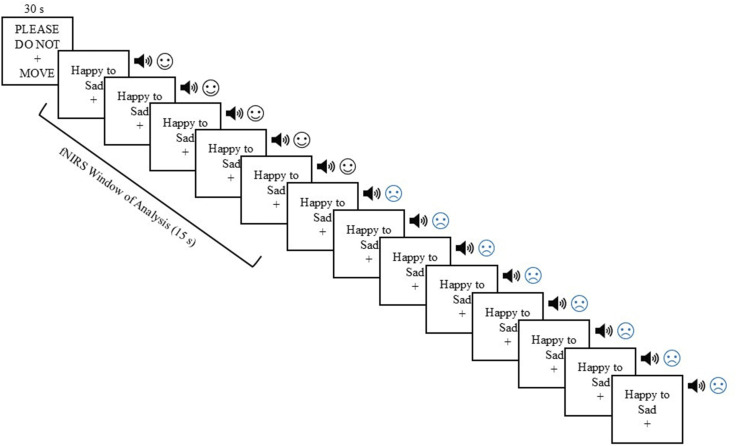
A schematic illustrating a single trial. Each trial began with 30 seconds of silence. Followed by the simultaneous presentation of visual and auditory stimuli. Visually, a fixation cross and text indicating the emotion transition (e.g., “Happy to Sad” in the happy–sad block) was presented. Auditorily, participants heard a sequence of speech samples from the RAVDESS. The sequence began with 5-12 presentations of speech spoken in an initial emotion (e.g., Happy) and then switched to presentations of a second emotion (e.g., Sad). The second emotion was played for up to 8 presentations or until the participant provided a key response, indicating they detected a changed in emotional prosody

Speech emotion perception was measured by a computer task of four practice trials and 16 experimental trials, organized into four blocks of emotion pairs (i.e., happy-sad, sad-happy, angry-calm and calm-angry). Each trial began with 30 seconds of silence with an on-screen fixation cross and instructions to remain still, allowing the hemodynamic response to return to baseline and for a baseline recording. Afterwards, participants viewed a fixation cross and heard a sequence of RAVDESS stimuli spoken by a given speaker. Each sequence began with stimuli of one emotion and after five to twelve stimuli presentations, the sequence changed to stimuli of the second emotion. Participants were asked to press the spacebar when they detected the change in emotion. The pseudorandom design of the emotion switching was to minimize participant daydreaming and motion artifacts from the key response during the fNIRS window of analysis which could have confounded the results. See Figure 2 for a schematic of a trial.

If no response was made after eight presentations of the second emotion, the trial ended and advanced to the next trial. Task performance was calculated as the number of additional presentations of the second emotion the participant heard before they responded, lower scores indicate better performance. Trials in which the participant responded before the emotion changed were removed. If the participant did not respond, then the trial was given the maximum score of 7. Participants were informed that their accuracy and response time was being recorded.

### Self-Report Measures

All self-report measures were selected based on wide use in the field and psychometric properties for generalizability in the field.

#### Demographics

Demographic information like age, sex, English proficiency, and general health and hearing were collected using self-report questions.

#### Cognitive Screening

Cognitive screening was performed using the Montreal Cognitive Assessment (MoCA), administered remotely in the videoconference format (i.e., MoCA Audiovisual). MoCA Audiovisual has been validated in several countries including Canada (Gagnon et al., 2022), demonstrating good internal reliability and construct validity (Carvalho et al., 2024; Iiboshi et al., 2020). The assessment was administered and scored by a trained and certified researcher. The standard cut-off score of at least 26 was used as the inclusion criterion.

#### Hearing-Aid Use

The Satisfaction with Amplification in Daily Life scale (SADL) assessed participants’ level of satisfaction with their hearing aids. Participants rated their satisfaction on each of the 15 items (e.g., *“How natural is the sound from your hearing aids?”*) using a 7-point Likert scale from 1 (*“Not at all”*) to 7 (*“Tremendously”*). A global mean score of at least 4 (indicating a *“Medium”* level of satisfaction), reported daily use of at least 4-8 hours a day and lifetime experience of at least 1 year were used as inclusion criteria.

#### Tinnitus Handicap Inventory (THI)

Due to the high comorbidity of hearing loss and tinnitus, the THI was used as a prescreening measure to minimize the effects of tinnitus over hearing loss. Across 25 items, the THI measures the impact of tinnitus on participants’ daily lives (Newman et al., 1996). Participants responded to each item (e.g., *“Because of your tinnitus, do you have trouble falling to sleep at night?”*) using a forced-choice format: 4 = *“Yes”*, 2 = *“Sometimes”* and 0 = *“No”*. The total score was categorized into one of five handicap levels, ranging from *“Slight or no handicap (Grade 1)”* to *“Catastrophic handicap (Grade 5)”* (McCombe et al., 2001). In a sample of adults (ranging from 19 to 84 years old), the THI showed valid factor structure and excellent internal reliability (Gos et al., 2020). A score of 36 or below, corresponding to *“Mild handicap (Grade 2)”*, was used as the eligibility threshold.

#### Toronto Alexithymia Scale (TAS-20)

Alexithymia, defined as difficulty identifying and describing one’s own emotions, was assessed using the 20-item TAS-20. Each item (e.g., *“I have feelings that I can’t quite identify”*) is rated on a 5-point Likert scale from 1 (“strongly disagree”) to 5 (“strongly agree”) to calculate a total score. Total scores were categorized into either no, possible or presence of alexithymia. A review of the psychometric literature over a 25-year period found that the TAS-20 demonstrates reliability (internal consistency and retest) and construct validity (factor analysis, convergent and discriminant validity; Bagby et al., 2020). A score of 61 or more (indicating presence of alexithymia) was used as exclusion criteria.

### fNIRS Data Acquisition and Preprocessing

HbO (and HbR) was recorded at 75 Hz using two Brite24 continuous-wave NIRS devices to form the Dual Brite system (Artinis Medical Systems, Netherlands). The probe consisted of 41 long channels (16 detectors and 16 light sources emitting two wavelengths, 760 nm and 850 nm) and four short-separate channels (SSCs). Long channels were formed by source-detector optode pairs to record neuronal activity approximately at the midway point of the optical path. The distance between a source-detector pair influences the depth the NIR light penetrates. In adults, the optimal source-detector pair distance is approximately 3 cm and records 1.5 to 2 cm into the cortex (Pinti et al., 2020). For short-separation channels (i.e., SSCs), the source-detector pair is placed closer together (1 to 1.5 cm) to record a shallower depth, not including the cortex. As a result, SSCs should only capture extracerebral components and physiological noise which can be regressed out from long channel signals to isolate cortical activity and improve data quality (Zhou et al., 2020).

**Figure 3.**
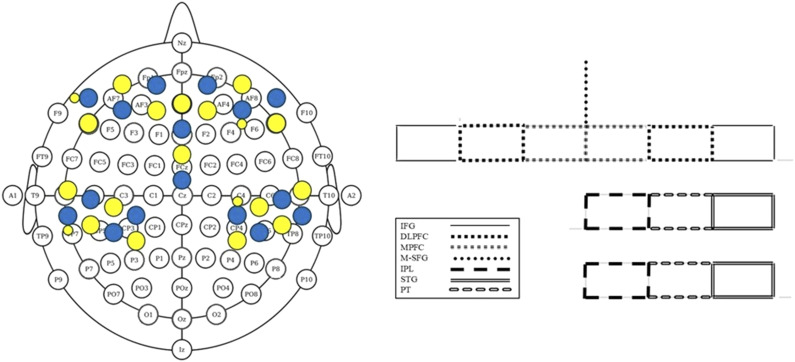
Illustrations of the fNIRS montage. Left: Optode montage. Light source and detector optodes are represented by light (yellow) and dark (blue) circles, respectively. Short-separation channels are depicted as a dark (blue) circle adjacent to a smaller light (yellow) circle. Right: Homer3 montage, illustrating channel clusters for each ROI

The cap was positioned with the Cz coordinate centered at the midpoint between inion-nasion and midpoint of the preauricular points. The NIRS devices were connected through Bluetooth to a Windows acquisition laptop running the recording software, OxySoft (version 3.5.15). Participants wore one of four cap sizes (small, medium, large and extra-large). The montage covers bilateral frontal (i.e., IFG, DLPFC, MPFC, medial-SFG), temporal (i.e., STG, PT) and parietal (i.e., IPL) cortical areas. See Figure 3 for an illustration of the montage. After each session, the optode positions were digitized using a 3D digitizer (Patriot, Polhemus, Colchester, VT) and projected onto the cortical surface to obtain MNI coordinates of the channels with AtlasViewer (Aasted et al., 2015). See Supplemental Material 3 for the sample-averaged MNI coordinates of each channel.

Data from OxySoft were saved in *.oxy5* format and was converted to the universal *.snirf* file format before being subjected to the following processing pipeline adapted from Zhou et al. (2020). Asterisks denote when Homer3 scripts were used (Huppert et al., 2009).

**1. Removal of step-like noise in light intensity data.**Step-like noise, from sudden loss of contact between optodes and skin or obstruction from hair, was removed by taking the derivative of each channel’s time series and setting absolute values exceeding two standard deviations from the mean to zero. The time series was then recovered by calculating the cumulative sum (i.e., integral) of the modified derivative.
**2. Exclude poor quality channels.** Raw light intensity data was assessed for quality using QT-NIRS; a software that bandpass filters the data between 0.5 and 2.5 Hz to isolate the heartbeat signal from both fNIRS wavelengths and correlates the two heartbeat signals. The correlation is represented by scalp coupling index (SCI) and peak spectral power (PSP) with higher values indicating better quality. QT-NIRS default parameters of SCI ≥ 0.8 and PSP ≥ 0.1 were used (Hernandez & Pollonini, 2020). Channels were considered poor quality if the quality threshold was below 60 (indicating that more than 60% of the timeseries falls below SCI and PSP thresholds).
3. Convert light intensity to optical density (OD)*. Refer to Huppert et al. (2009) for details.
4. Correct motion artifacts*. Motion artifacts (e.g., spikes) were corrected through wavelet decomposition where wavelet coefficients exceeding 1.5 times the interquartile range were set to zero.
5. Convert OD to hemoglobin concentration using the Modified Beer—Lambert law*. Refer to Huppert et al. (2009) for details.
6. Bandpass filter (0.01 – 1.5 Hz)*. A bandpass filter was applied to the fNIRS signal to remove low- (e.g., drift) and high-frequency noise (e.g., respiration).
**7. Short-channel correction:** Each channel underwent SSC correction by fitting a general linear model to each channel’s fNIRS signal using the time series of the nearest SSC (based on Euclidean distance) as the regressor. The physiological noise captured by the SSC was regressed out of each channel’s fNIRS signal by subtracting the portion of the signal predicted by the SSC from the channel’s signal.
8. Bandpass filter (0.01 – 0.09 Hz)*. Remaining physiological noise (e.g., heart rate or motion artifacts due to high-frequency noise bursts not removed through wavelet decomposition) was removed with a bandpass filter.

#### fNIRS Block Average

The hemodynamic response was defined as 15 seconds following the start of each trial and was baseline corrected using the average 5-second baseline preceding each trial. Following preprocessing, HbO was extracted for each trial and channel, then averaged across time. If the participant provided a key press during the fNIRS window of analysis, then the HbO of that trial was removed.

#### fNIRS Connectivity

After preprocessing, the HbO time series during the task was extracted, defined as the period from the onset of the first trial to the end of the last trial. The time series was then subjected to the following connectivity analysis pipeline adapted from Chen et al. (2017):

**1. Prewhiten.** A prewhitening filter was defined from an autoregressive (AR) model (Santosa et al., 2017). The best fitting AR model for each channel’s time series was selected based on the order that minimized the Bayesian information criteria. This AR was then applied as a filter using MATLAB’s built-in filter function (*filter.m*) to remove autocorrelation (i.e., temporal dependence).
**2. Correlation Matrix.** A Pearson correlation coefficient was computed for all possible channel pairs.
**3. Fisher Transformation.** All Pearson correlation coefficients were converted to a z value using Fisher z-transformation so that the data was normally distributed and can be subjected to subsequent statistical analyses.
**4. Remove Anticorrelated Relationships.** Only positive connections (i.e., z-values) between channels were retained for better reliability (Noble et al., 2019; Shehzad et al., 2009) and interpretability.
**5. Node Pair Averaging.** The z-values were averaged across all channel pairs that correspond to node pairs (i.e., edges) within a given network for each hemisphere separately as averaging within networks has greater reliability than individual edges (Rai et al., 2025; Tozzi et al., 2020).

### Statistical Analysis Plan

Analyses were conducted in R version 4.4.2 with the following packages: *DHARMa* (0.4.7), *effectsize* (1.0.0), *emmeans* (1.10.6), *glmmTMB* (1.1.11), *lmtest* (0.9.40), *lme4* (1.1.35.5), *lmerTest* (3.1.3) and *psych* (2.4.6.26). For all continuous linear models, outliers were defined as values exceeding 1.5 times the interquartile range and removed separately for each combination of predictors (i.e., group and hemisphere). For discrete response models, a simulation-based method for identifying the presence of outliers were conducted using *DHARMa*; no outliers were identified. All linear mixed models included a random intercept per participant and a binary predictor of group with normal hearing or hearing aided as the reference group, depending on the comparison. Effect sizes were calculated as partial eta-squared for linear models and as incidence rate ratios (IRR) for discrete models.

Group differences in task performance were tested with three separate generalized linear mixed models (GLMMs) to compare: i) normal hearing vs. hearing loss aided, ii) normal hearing vs. hearing loss unaided and iii) hearing loss aided vs. unaided listening. Following prior research, emotion was not a factor; models controlling for emotion showed the same pattern of results. GLMMs were used to analyze the discrete dependent variable (i.e., the number of additional presentations of the second emotion heard before providing a response) and to account for the clustered data structure from the repeated measures design, as participants provided a response for each trial. A Poisson distribution was used for the normal hearing vs. hearing loss aided comparison whereas a negative binomial type II distribution was used for the remaining comparisons due to overdispersion detected in the data (Too et al., 2025).

Group differences in activation within ROIs were assessed using HbO concentration in micromolar units. To enhance signal robustness, HbO data were averaged across all channels within each ROI, with left and right hemispheres treated separately when applicable (Wiggins et al., 2016; see Figure 3 for channel groupings). Each ROI was comprised of at least two channels, otherwise, that ROI was deleted for that participant. Overall, each participant contributed an HbO value (averaged across time) per trial for each ROI and when applicable, for each hemisphere. To account for the clustered data structure, linear mixed-effects models (LMMs) were used. Based on prior research in normal-hearing adults, neural activation in some ROIs could be bilateral or lateralized to either hemisphere, but findings are mixed. Therefore, a data-driven, nested modeling approach was used to determine which LMM best fits the data, allowing us to explore potential interactions between group and hemisphere. Specifically, each ROI was fitted to two to four separate LMMs, when applicable: i) an intercept-only model, ii) fixed effect of group model, iii) fixed effects of group and hemisphere model and iv) interaction between group and hemisphere model. Model comparisons were evaluated using likelihood ratio tests (Barr et al., 2013). The fixed effects of the best-fitting model for each ROI were corrected for multiple comparisons using false discovery rate (Benjamini & Hochberg, 1995).

Task-based functional connectivity for each node pair of interest was analyzed using a 2×2 ANOVA to examine the main effect of group while controlling for hemisphere, as connectivity was not expected to differ between hemispheres. Hemisphere (left vs. right) was always the within-group factor and Group was the between- or within-subjects factor depending on the comparison: normal hearing vs. hearing loss aided (between); normal hearing vs. hearing loss unaided (between); and hearing loss aided vs. unaided listening (within).

## Results

### Speech Emotion Perception Task Performance

**Figure 4.**
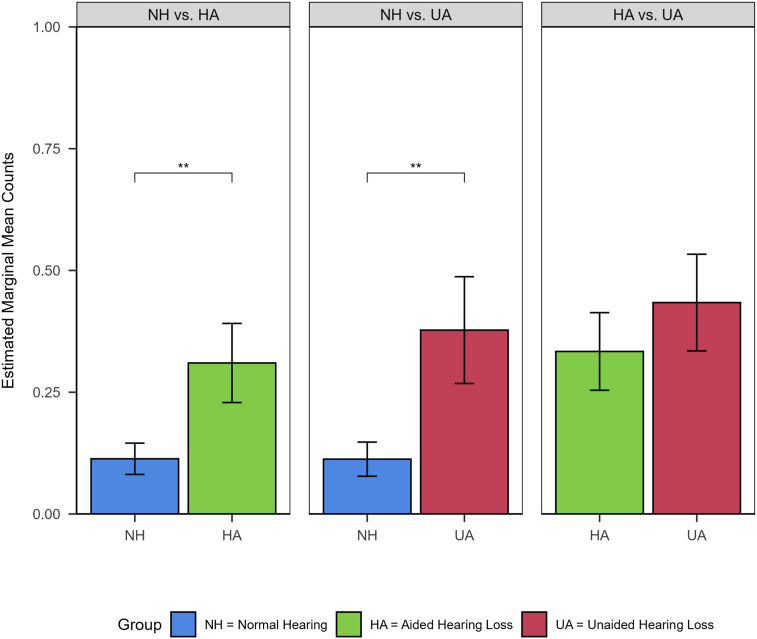
Estimated marginal means of performance on the speech emotion perception task, reported on the response scale. Raw group means are reported in text. Lower scores indicate better task performance. Error bars represent the standard error. *p < .01

Aligned with our hypotheses, there was a significant main effect of group on task performance (*IRR* = 2.74, *p* = .007) such that compared to normal hearing, those with hearing loss under aided listening heard almost three times (174% increase) as many stimuli after the emotion changed before responding. Under unaided listening, those with hearing loss heard over three times (235% increase) as many stimuli than the normal-hearing group before indicating a change in emotion occurred, *IRR* = 3.35, *p* = .003. Contrary to our hypothesis, but aligned with the broader literature, there was no significant difference in task performance between aided and unaided listening for those with hearing loss, *IRR* = 1.30, *p* > .05. See Figure 4.

### Group Differences in ROIs

As hypothesized, models including group as a fixed effect provided a significantly better fit than the intercept-only model for specific ROIs. These regions—the IPL, PT and medial-SFG—have been implicated in integrative processes, auditory processing and top-down control processes, respectively. Model fit indices for between-group comparisons are provided in Table 2 and Table 3, while within-group comparisons are presented in Table 4. No other ROIs showed a significant improvement in model fit (all *p* > .065).

**Table 2.** Nested Model Performance for the Effect of Group, Comparing Normal Hearing (Reference Group) and Aided Hearing Loss on Oxygenated Hemoglobin in Regions of Interest

​	Fixed effects									Goodness of fit and comparison			
Model	Est.	*β*	SE	df	t	*p*	*p-adj*	95%CI [UL, LL]	*η*_*p*_^*2*^	*χ* ^*2*^	AIC	df	*p*
Planum Temporale													
Baseline													
Intercept	-0.32	-.003	0.54	28.72	-0.60	.55	-	-1.42, 0.76	-	​	5952.0	​	​
+ Main effect													
Intercept	-1.39	-.10	0.62	27.82	-2.23	**.03**	-	-2.65, -0.12	-	6.14	5947.9	1	**.01**
Group	2.56	.23	0.96	26.82	2.66	**.01**	**.01**	0.58, 4.49	.21	​	​	​	​
Inferior Parietal Lobe													
Baseline													
Intercept	0.25	-.006	0.90	26.78	0.28	.78	-	-1.61, 2.07	-	​	6183.0	​	​
+ Main effect													
Intercept	-2.03	-.14	0.92	25.85	-2.21	**.04**	-	-3.90, -0.16	-	11.91	6173.1	1	***
Group	5.69	.34	1.43	25.73	3.99	***	***	2.74, 8.55	.38	​	​	​	​

*Note. β* = standardized beta coefficient, UL = upper limit, LL = lower limit, AIC = Akaike information criterion, *p-adj = FDR-corrected p-values,* *** *p* < .001.

**Table 3.** Nested Model Performance for the Effect of Group, Comparing Normal Hearing (Reference Group) and Unaided Hearing Loss on Oxygenated Hemoglobin in Regions of Interest

​	Fixed effects									Goodness of fit and comparison			
Model	Est.	*β*	SE	df	t	*p*	*p-adj*	95%CI [UL, LL]	*η*_*p*_^*2*^	*χ* ^*2*^	AIC	df	*p*
Planum Temporale													
Baseline													
Intercept	-0.43	-.0002	0.50	28.27	-0.85	.40	-	-1.45, 0.59	-	​	6405.5	​	​
+ Main effect													
Intercept	-1.39	-.09	0.62	29.29	-2.23	**.03**	-	-2.65, -0.12	-	4.82	6402.7	1	**.03**
Group	2.13	.19	0.93	27.22	2.30	**.03**	**.03**	0.25, 4.01	.16	​	​	​	​
Medial Superior Frontal Gyrus													
Baseline													
Intercept	5.87	.006	3.09	14.13	1.90	.08	-	-0.54, 12.47	-	​	1897.4	​	​
+ Main effect													
Intercept	-0.19	-.17	3.34	14.68	-0.06	.95	-	-7.12, 6.87	-	6.07	1893.3	1	**.01**
Group	13.89	.41	5.11	14.33	2.72	**.02**	**.03**	3.26, 24.72	.34	​	​	​	​

*Note. β* = standardized beta coefficient, UL = upper limit, LL = lower limit, AIC = Akaike information criterion, *p-adj = FDR-corrected p-values,* *** *p* < .001.

**Table 4.** Nested Model Performance for the Effect of Group, Comparing Aided (Reference Group) and Unaided Hearing Loss on Oxygenated Hemoglobin in Regions of Interest

​	Fixed effects									Goodness of fit and comparison			
Model	Est.	*β*	SE	df	t	*p*	*p-adj*	95%CI [UL, LL]	*η*_*p*_^*2*^	*χ* ^*2*^	AIC	df	*p*
Inferior Parietal Lobe													
Baseline													
Intercept	1.81	-.01	0.85	10.05	2.12	.06	-	-0.13, 3.56	-	​	5276.2	​	​
+ Main effect													
Intercept	3.50	.10	1.02	27.98	3.43	**.002**	-	1.28, 5.51	-	6.08	5272.1	1	**.01**
Group	-3.12	-.20	1.25	618.27	-2.50	**.01**	**.01**	-5.59, -0.64	.01	​	​	​	​
Medial Superior Frontal Gyrus													
Baseline													
Intercept	-.87	-.02	3.82	12.18	-0.23	.82	-	-9.25, 7.03	-	​	2276.4	​	​
+ Main effect													
Intercept	-9.29	-.22	4.31	19.32	-2.16	**.04**	-	-18.51, -0.61	-	16.65	2261.8	1	***
Group	24.57	.60	5.90	139.18	4.16	***	***	12.85, 36.58	.11	​	​	​	​

*Note. β* = standardized beta coefficient, UL = upper limit, LL = lower limit, AIC = Akaike information criterion, *p-adj = FDR-corrected p-values,* *** *p* < .001.

**Figure 5.**
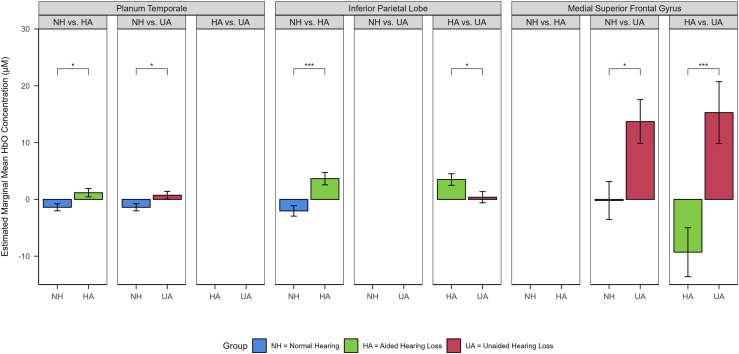
Estimated marginal means of HbO during the speech emotion perception task. Raw group means are reported in text. Group comparisons are left blank in the cases where the model including the effect of Group did not provide a better fit than the null (intercept-only) model. Error bars represent the standard error. FDR-corrected significance values are depicted. *p < .05, ***p < .001

**Figure 6.**
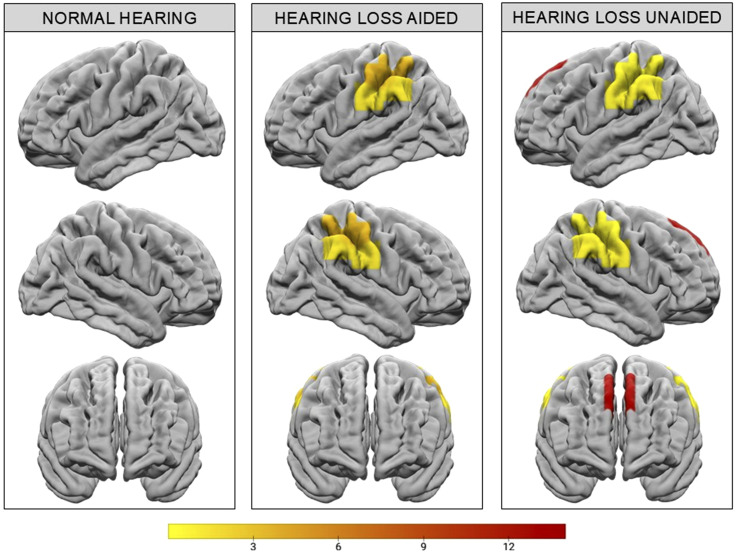
Brain activation patterns during the speech emotion perception task. Data are presented as raw group means, with negative group means set to zero. Shaded areas indicate the magnitude of the increased activation (HbO) relative to baseline

Compared to normal hearing, those with hearing loss during aided listening showed greater HbO in the IPL, *M(SE)*_*NH*_ = -2.03 (0.92), *M(SE)*_*HA*_ = 3.66 (1.09), and PT, *M(SE)*_*NH*_ = -1.39 (0.62), *M(SE)*_*HA*_ = 1.17 (0.73). During unaided listening, there was greater HbO in the medial-SFG, *M(SE)*_*NH*_ = -0.19 (3.34), *M(SE)*_*UA*_ = 13.70 (3.87) and PT *M(SE)*_*NH*_ = -1.39 (0.62), *M(SE)*_*UA*_ = 0.73 (0.68) relative to normal hearing. Within the hearing loss group, aided listening showed greater HbO in IPL, *M(SE)*_*HA*_ = 3.50 (1.02), *M(SE)*_*UA*_ = 0.38 (0.98), whereas unaided listening had greater HbO in medial-SFG than aided listening, *M(SE)*_*HA*_ = -9.29 (4.31), *M(SE)*_*UA*_ = 15.28 (5.45). Notably, effects involving the medial-SFG were consistently larger in magnitude than those observed in IPL or PT, as reflected in strikingly larger regression coefficients. For model statistics, see Table 2andTable 3 for between-group comparisons and Table 4 for within-group comparisons. Results are illustrated in Figure 5 and in Figure 6 as group mean brain activation patterns.

### Group Differences in Task-Based Functional Connectivity

Controlling for hemisphere, there was lower IFG–IPL connectivity in the hearing loss group during aided listening (*M* = .12, *SD* = .066) compared to normal-hearing group (*M* = .19, *SD* = .14). Within the hearing loss group, IFG-IPL connectivity was also lower during aided listening (*M* = .10, *SD* = .044) compared to unaided listening (*M* = .17, *SD* = .07). See Table 5 for model statistics.

**Table 5.** Analysis of Variance Table for the Effect of Group on Task-Based Functional Connectivity Between Inferior Frontal Gyrus and Inferior Parietal Lobe, Controlling for Hemisphere

Predictor	Sum of squares	Mean square	Numerator *df*	Denominator *df*	*F*	*p*	*η*_*p*_^*2*^
Normal Hearing (reference group) versus Aided Hearing Loss							
Group	0.06	0.06	1	26.93	4.33	**.047**	.14
Hemisphere	0.002	0.002	1	27.44	0.14	.71	.00
Normal Hearing (reference group) versus Unaided Hearing Loss							
Group	0.0004	0.0004	1	54	0.026	.87	.00
Hemisphere	0.0008	0.0008	1	54	0.058	.81	.00
Aided (reference group) versus Unaided Hearing Loss							
Group	0.04	0.04	1	26.57	15.90	***	.37
Hemisphere	0.02	0.02	1	25.10	6.13	**.02**	.20

*Note.* *** *p* < .001.

**Figure 7.**
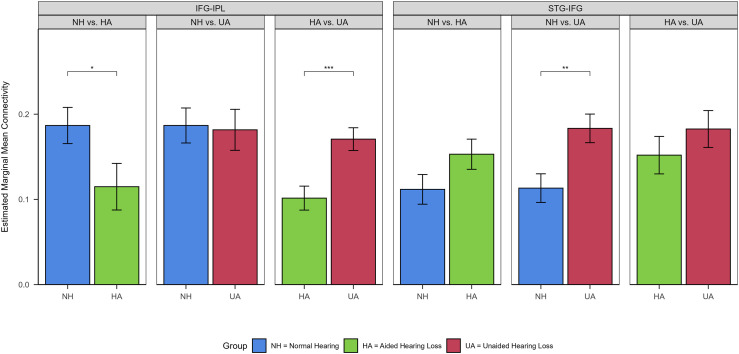
Estimated marginal means of functional connectivity during the speech emotion perception task while controlling for hemisphere. IFG-IPL displays connectivity between inferior frontal gyrus and inferior parietal lobe while STG-IFG displays connectivity between superior temporal gyrus and inferior frontal gyrus. Error bars represent the standard error. *p < .05, ** p < .01, *** p < .001

**Figure 8.**
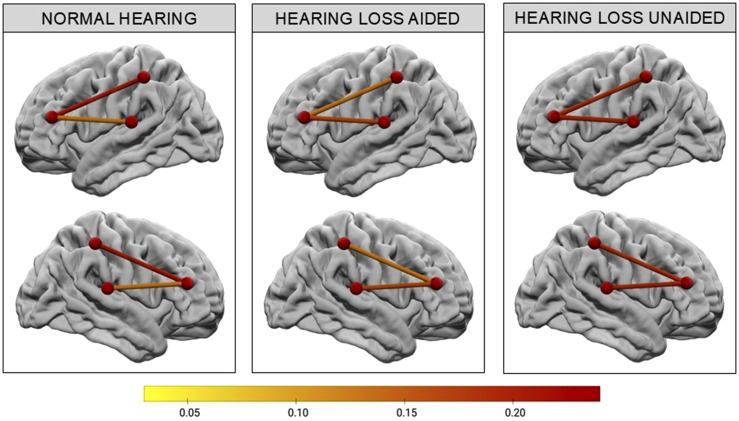
Task-based functional connectivity patterns during the speech emotion perception task. Data are presented as raw group means. Edge color indicates the magnitude of the connectivity

Regardless of hemisphere, the connectivity between STG-IFG was higher for the unaided hearing loss group (*M* = .18, *SD* = .087) than the normal-hearing group (*M* = .11, *SD* = .06). There was no difference in STG-IFG connectivity within the hearing loss group during aided (*M* = .15, *SD* = .02) versus unaided listening (*M* = .18, *SD* = .02). See Table 6 for model statistics. Results are illustrated in Figure 7 and in Figure 8 as brain connectivity patterns. The main effect of group while controlling for hemisphere in all other models was non-significant (all *p* > .10).

**Table 6.** Analysis of Variance Table for the Effect of Group on Task-Based Functional Connectivity Between Superior Temporal Gyrus and Inferior Frontal Gyrus, Controlling for Hemisphere

Predictor	Sum of squares	Mean square	Numerator *df*	Denominator *df*	*F*	*p*	*η*_*p*_^*2*^
Normal Hearing (reference group) versus Aided Hearing Loss							
Group	0.02	0.02	1	48	2.77	.10	.05
Hemisphere	0.02	0.02	1	48	2.42	.13	.05
Normal Hearing (reference group) versus Unaided Hearing Loss							
Group	0.05	0.05	1	25.04	8.74	**.007**	.26
Hemisphere	0.00	0.00	1	25.84	0.006	.94	.00
Aided (reference group) versus Unaided Hearing Loss							
Group	0.01	0.01	1	37.61	1.47	.23	.04
Hemisphere	0.06	0.06	1	34.92	8.00	**.008**	.19

*Note.* *** *p* < .001

### Brain-Behaviour Relationships

Exploratory analyses examined correlations between neural activity (localized activity and functional connectivity) and task performance within each group. All correlations were *p* > .05.

## Discussion

Hearing loss in older adults has been associated with deficits in speech emotion perception and the absence of a clear benefit from hearing aids remains poorly understood. To address this gap, we combined behavioural measures with fNIRS to examine the neural processing of emotional speech in older adults with and without hearing loss, during aided and unaided listening conditions. Consistent with previous work, normal-hearing older adults outperformed those with hearing loss, regardless of hearing-aid use, requiring fewer additional presentations of the second emotion to detect a change. There was no statistically significant difference between aided and unaided listening in the number of presentations needed to detect a change. Although not statistically significant, clinically relevant differences cannot be ruled out given that aided listening reduced the number of required presentations by approximately 30% relative to unaided listening. Critically, our fNIRS results extend the behavioural findings by revealing hearing loss and hearing aid related differences in both regional activation and task-based functional connectivity, providing a foundation for a neurocognitive account of the consistent perceptual deficits observed in prior work.

### A Neurocognitive Account of the Perceptual Deficits

In our framework, increased PT activity reflects greater demands on early auditory analysis, IPL engagement reflects higher-order integration when partial signal restoration is available, and medial-SFG recruitment reflects top-down support under maximal signal degradation. Together, we view the activation and connectivity findings as suggestive of an increased reliance on higher-order and frontal control processes as the auditory signal becomes progressively degraded.

Consistent with this account, group differences in neural activation emerged across several regions of interest. Individuals with hearing loss showed greater PT activity than those with normal hearing, regardless of hearing-aid use, indicating increased demands on early auditory processing. In contrast, IPL activity was greatest during aided listening in individuals with hearing loss, whereas medial-SFG activity was most pronounced during unaided listening.

Because our analyses adopted a data-driven approach, the best-fitting linear mixed-effects models included only a main effect of group and did not retain hemisphere as a predictor. This may reflect either minimal hemispheric asymmetries for the processes under study or limited power to detect more subtle lateralized effects. Accordingly, the reported findings reflect group differences in regional activation without distinguishing between left and right homologous regions.

The PT is involved in early auditory processing (Binder et al., 1996) and acts as a computational hub to process spectrotemporally complex sounds (Griffiths & Warren, 2002; Rauschecker & Scott, 2009). This profile makes the PT well suited for supporting speech emotion perception given that speech and vocal emotion are characterized by dynamic changes in frequency across time. Broadly, the PT has shown greater engagement during active listening of tones and words compared to passive listening (Binder et al., 1996). More specifically, recent fMRI work has linked the PT to active speech emotion perception. In normal-hearing young adults, fMRI captured greater PT activity when listeners categorized emotional and neutral speech based on its emotional content rather than its grammatical structure (Beaucousin et al., 2011). In the present study, individuals with hearing loss exhibited greater PT oxygenation than normal-hearing listeners irrespective of hearing-aid use, suggesting sustained reliance on early auditory analysis when the acoustic signal is degraded. Although hearing aids improve audibility, they do not fully restore to normal hearing. For instance, distortion of sounds due to hearing loss, such as loss of fine-frequency tuning (i.e., spectral smearing) is not resolved by amplification through hearing-aids (see Lesica, 2018 for a review). These deficits in suprathreshold processing even with hearing aids may explain why elevated PT recruitment persisted even under aided listening.

In contrast, the IPL is implicated in higher-order integration of complex auditory information, including spatial and emotional aspects of sound (Rauschecker, 2011; Rauschecker & Scott, 2009; Shamay-Tsoory, 2011). We found that IPL activity was greatest during aided listening in individuals with hearing loss, suggesting that partial restoration of the acoustic signal may be sufficient to engage higher-level integrative processes, even if perceptual performance remains impaired. During unaided listening, however, this parietal engagement was reduced, and greater recruitment of frontal regions was observed instead.

Specifically, medial-SFG activity was strikingly elevated during unaided listening in individuals with hearing loss (see Figure 5). However, these findings should be interpreted cautiously because the medial-SFG analyses were based on a smaller sample than the other ROIs, as anatomical variability often reduced optode contact in this region and increased channel rejection. The elevated medial-SFG activity may reflect increased vigilance. In an fMRI study of normal-hearing young adults, the SFG was more active when participants paid attention to the valence of adjectives than during passive listening (Wegrzyn et al., 2017). More specifically, it could reflect increased activity in the cingulo-opercular network (COPN) sometimes referred to as the salience network. Broadly, the COPN is engaged during identification tasks when listeners detect that their response may be incorrect, consistent with a performance-monitoring (error-monitoring) role. Evidence suggests that when the COPN detects an uptick in errors, it marshals higher-order cognitive processes to support task performance (Menon, 2011). Consistent with this, in adults with hearing loss, elevated COPN activity on a preceding trial predicted greater likelihood of correct word recognition on the subsequent trial (Vaden et al., 2016). Accordingly, the COPN has been proposed to be a domain-general brain network to support perception of acoustically degraded speech like in cases of hearing loss (see Peelle, 2018 for a review). These insights from studies of word perception may extend to the current study because the stimuli consisted of emotionally prosodic speech, even though the task emphasized emotion change detection rather than word recognition. The medial-SFG has been proposed to be part of the COPN given its anatomical proximity immediately superior to the ACC, a central node of this network (e.g., Dosenbach et al., 2008; Menon & D’Esposito, 2022). Taken together, increased medial-SFG recruitment in older adults during unaided listening likely reflects greater top-down engagement of frontal control processes to support emotion perception when bottom-up auditory information is most impoverished.

Task-based functional connectivity results further support this interpretation. Individuals with hearing loss under unaided listening showed greater connectivity between STG and IFG than normal-hearing listeners, consistent with increased top-down integration when bottom-up auditory cues are degraded. In hierarchical models of auditory processing, sensory regions project to higher-order frontal areas as task demands increase (Rauschecker & Scott, 2009). Within this framework, partial restoration of the acoustic signal via hearing aids would be expected to reduce reliance on such top-down pathways. Although group differences in STG–IFG connectivity did not reach statistical significance, likely due to substantial interindividual variability, the observed pattern of group means was consistent with this prediction, suggesting partial normalization of network dynamics under aided listening.

Older adults with hearing loss showed the weakest task-based functional connectivity between IFG and IPL during aided listening. Connectivity between IFG-IPL reflects sensorimotor mapping, with posterior STG regions, including the PT, projecting dorsally to the IPL to integrate sensory input from the PT with motor plans from the IFG (Rauschecker & Scott, 2009). This sensorimotor mapping is consistent with internal models, where external auditory input is continuously mapped onto motor representations to support perception through predictive coding (Rauschecker, 2011). Sensorimotor mapping has also been implicated in prosody perception (Sammler et al., 2015) and emotion perception models where IFG-IPL interactions internally emulate others’ emotional states, activating corresponding limbic regions and enabling shared affective experiences (Prochazkova & Kret, 2017; Yu & Chou, 2018). The weakened IFG-IPL connectivity under aided listening suggests less sensorimotor integration despite increased localized IPL activity. While hearing aids partially restore auditory signals, shifting speech emotion perception to rely more on sensorimotor areas like IPL instead of medial-SFG, this shift is ineffective given the reduced IFG-IPL connectivity and no significant behavioural benefit. We speculate that signal processing from hearing aids may make the auditory signal too artificial, reducing one’s ability to map the external auditory input onto one’s internal motor system. Future studies could simulate hearing aids in normal-hearing participants and systematically vary the intensity of the signal processing to assess changes in IFG-IPL connectivity during speech emotion perception. This research may inform development of interventions that can be used alongside traditional auditory rehabilitation devices. For example, imitation-based training has improved imitation and increased engagement of sensorimotor systems in autistic children (Datko et al., 2018) and could be adapted to support hearing-aid users. While hearing aids partially improve the auditory signal, they also limit the sensorimotor integration involved in speech emotion perception.

There were no significant correlations between neural activity and task performance within each group, which may reflect limited statistical power and restricted range in values when each group was examined separately. We also found no group differences in task-based functional connectivity between the IPL and DLPFC of the FPN. Since the FPN is engaged in cognitively demanding tasks, perhaps group differences may arise if the task was more cognitively demanding. Older adults with hearing loss may recruit cognitive networks or brain areas for speech emotion processing in more difficult or ambiguous environments. For instance, Alba-Ferrara et al. (2011) found that relative to simple emotions (i.e., happy, sad, angry), more complex emotions (i.e., proud, guilty, bored) engaged more of the IFG and MPFC. Future research could use more ambiguous speech emotion stimuli by adding background noise to assess differential engagement of cognitive brain networks.

### Why Do Hearing Aids Ultimately Fail to Confer Benefit

One explanation for the limited behavioural benefit of hearing aids in speech emotion perception is that signal processing strategies optimized for audibility and intelligibility may inadvertently distort acoustic cues critical for conveying emotion. Although amplification is designed to restore audibility, gain is applied unevenly across frequencies according to an individual’s hearing profile and is accompanied by nonlinear amplitude compression to prevent discomfort. Because emotional expressions in speech rely heavily on spectral cues, such frequency-dependent amplification and compression may distort emotionally relevant information. Consistent with this account, Buono et al. (2021) showed that removing either high- or low-frequency content from nonspeech emotion sounds altered the spectral centroid and attenuated valence and arousal ratings even among normal-hearing listeners. Similarly, spectral centroid has been identified as an acoustic cue for arousal and valence of vocal emotion (Sauter et al., 2010). As a result, the nonlinear amplification across frequencies may alter the spectral balance, alter acoustic cues to emotion and consequently vocal emotion perception. While modern hearing aids offer environment-specific programs (e.g., quiet, noise, car), no standard processing strategy is tailored to emotion perception.

In addition to spectral balance, voice pitch is a key acoustic cue important for vocal emotion perception (Sauter et al., 2010). Although vocal pitch cues are conveyed primarily in lower frequencies and hearing loss is typically greater at higher frequencies, older adults with hearing loss nevertheless show impairments in vocal emotion perception relative to normal-hearing listeners. This paradox may reflect suprathreshold processing deficits that affect pitch perception despite preserved audibility at lower frequencies. Because hearing aids primarily compensate for attenuation rather than distortion of the auditory signal, these suprathreshold deficits are unlikely to be fully resolved through amplification alone (Lesica, 2018).

Alternatively, hearing aids may confer a modest benefit for speech emotion perception that is highly variable across individuals and therefore difficult to detect with the present sample size. For instance, in a sample of 141 children (55 hearing-aid users), hearing-aided children showed vocal emotion recognition difficulties at the group level, but at the individual level, over one-third performed above the 25^th^ percentile of the normal hearing children (Rachman et al., 2025). It is also possible that the change-detection paradigm used here, although well suited to capturing graded perceptual differences, lacked sufficient sensitivity to reveal small effects. Future work could address these limitations by adopting complementary assessment approaches, including ecologically valid methods like ecological momentary assessment, which has captured benefits of aided versus unaided listening on experienced valence and arousal in everyday contexts (Holman & Naylor, 2025). Hearing aids also have noise reduction algorithms to improve audibility in environments with competing background noise or speech. The stimuli used in this current study and within the literature are void of background noise which may not reflect a listening context in which aided versus unaided listening is beneficial for speech emotion perception. Similarly, the on-screen emotion transition cue (e.g., “Happy to Sad” in the happy–sad block) in our task could be removed as it may prime participants. Future research could include background noise and remove the on-screen text from our task to assess whether the benefit of hearing aids over unaided listening is present in more ambiguous listening contexts.

### Limitations and Future Directions

Several limitations should be considered when interpreting the present findings. First, the absence of a control task limits the ability to determine whether the observed behavioural and neural effects were specific to speech emotion perception or instead reflected more general auditory change-detection processes. However, this concern is partially mitigated by the use of multiple emotional intensities, sentences, and recordings from the RAVDESS, which introduced substantial variability across stimuli while preserving the emotional-change demands of the task. Moreover, participants generally required fewer than one additional presentation on average to detect an emotional change, suggesting that performance was meaningfully driven by emotion perception rather than by simple auditory novelty detection. Nevertheless, future work should incorporate comparison conditions (e.g., non-emotional auditory changes, intensity changes, or speaker changes) to more directly dissociate processes related to general auditory change detection from those specific to speech emotion perception.

A second limitation concerns the characterization of the normal-hearing group. Older adults were classified as having normal hearing based on pure-tone audiometric thresholds, although audiograms do not capture all aspects of auditory function. Relative to younger adults, older adults with clinically normal hearing can still exhibit suprathreshold processing deficits, including impairments in dynamic pitch processing (Russo et al., 2012; Shen et al., 2016), which are relevant for speech emotion perception. Consistent with this, age-related reductions in the use of F0 contour cues have been linked to poorer vocal emotion perception even among individuals with normal low-frequency hearing (Chatterjee et al., 2026). Accordingly, the present group differences may reflect both hearing-loss-related and broader age-related changes in suprathreshold auditory processing. Future studies could include younger normal-hearing adults to disentangle the effects of aging from those associated with hearing loss in older adults.

In the current study, participants wore their own hearing aids during testing to better reflect their real-world usage and the diverse range of devices worn by older adults. As presented in Supplemental Material 4, there was a range in the hearing aid styles, brands and models. Future work may explore whether technological differences in hearing aids impact speech emotion perception in older adult users.

## Conclusion

The present study addresses a critical gap by characterizing the cortical processes engaged during speech emotion perception in older adults with hearing loss, including during aided listening. By integrating behavioural performance with measures of localized activation and task-based functional connectivity, we demonstrate that hearing loss and hearing-aid use are associated with systematic shifts in neural processing strategies, marked by increased reliance on higher-order and frontal processes. These findings provide a neurocognitive explanation for why improved audibility does not necessarily translate to improved emotional communication and highlight the utility of fNIRS for studying affective speech processing in hearing-aid users.

## Supplemental Material

Supplemental Material - Hearing Aids Reshape Neural Processing of Emotional Speech Without Improving Emotion Perception Supplemental Material for Hearing Aids Reshape Neural Processing of Emotional Speech Without Improving Emotion Perception by Carmen Dang, Gurjit Singh, Frank A. Russo in Trends in Hearing

## Data Availability

The datasets generated during and/or analyzed during the current study are available from the corresponding author on request.*
