# Supplementary material for: Hearing Aids Reshape Neural Processing of Emotional Speech Without Improving Emotion Perception
Source: Trends Hear. 2026 Jul 2;30:23312165261465515. doi: 10.1177/23312165261465515 (PMC13329022; doi:10.1177/23312165261465515)
Supplement: Supplemental Material - Hearing Aids Reshape Neural Processing of Emotional Speech Without Improving Emotion Perception [file sj-pdf-1-tia-10.1177_23312165261465515.pdf]

Supplementary Material 1

L'Oreal Skin Color Chart

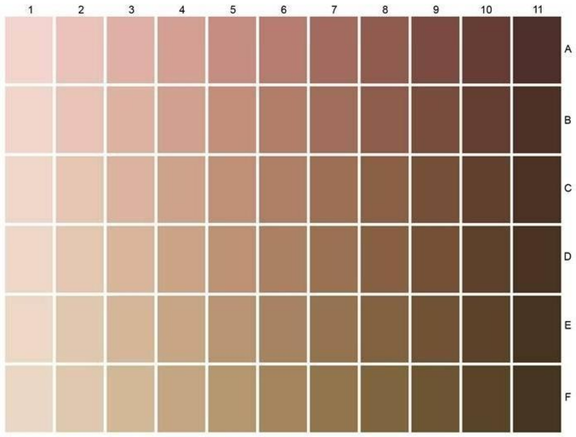

Andre Walker Hair Typing System

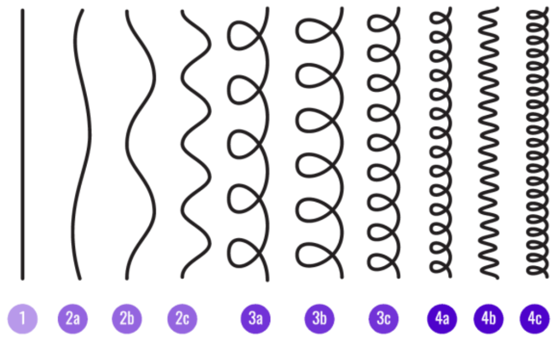

Skin Shade

	**1C**	**1D**	**1E**	**1F**	**2B**	**2C**	**2D**	**2E**	**2F**	**3B**	**3C**	**3D**	**3E**	**4E**
Hearing Aid Users	1	2	2	2	0	1	1	0	0	0	0	3	1	1
Normal Hearing	3	2	0	2	1	0	0	2	2	1	2	1	0	1

Hair Type

	**1**	**2A**	**2B**	**2C**	**3A**	**3C**	**4C**	**Bald**
Hearing Aid Users	4	3	3	3	0	0	0	1
Normal Hearing	7	4	2	0	2	1	1	0

Supplementary Material 2

Participant	Group	Sound Level (dB(A))
Anonymized_01	Normal Hearing	62.33
Anonymized_02	Normal Hearing	60.73
Anonymized_03	Normal Hearing	62.33
Anonymized_04	Normal Hearing	45.4
Anonymized_05	Normal Hearing	61.43
Anonymized_06	Normal Hearing	66.8
Anonymized_07	Normal Hearing	53.92
Anonymized_08	Normal Hearing	59.7
Anonymized_09	Normal Hearing	46.6
Anonymized_10	Normal Hearing	59.03
Anonymized_11	Normal Hearing	53.92
Anonymized_12	Normal Hearing	59.03
Anonymized_13	Normal Hearing	53.92
Anonymized_14	Normal Hearing	53.92
Anonymized_15	Normal Hearing	59.03
Anonymized_16	Normal Hearing	59.03
Anonymized_17	Normal Hearing	65.36
Anonymized_18	Hearing Loss (Aided)	61.43
Anonymized_19	Hearing Loss (Aided)	62.33
Anonymized_20	Hearing Loss (Aided)	59.03
Anonymized_21	Hearing Loss (Aided)	72.64
Anonymized_22	Hearing Loss (Aided)	53.92
Anonymized_23	Hearing Loss (Aided)	65.36
Anonymized_24	Hearing Loss (Aided)	62.33
Anonymized_25	Hearing Loss (Aided)	65.36
Anonymized_26	Hearing Loss (Aided)	65.36
Anonymized_27	Hearing Loss (Aided)	59.03
Anonymized_28	Hearing Loss (Aided)	59.03
Anonymized_29	Hearing Loss (Aided)	67.6
Anonymized_30	Hearing Loss (Aided)	65.36
Anonymized_31	Hearing Loss (Aided)	62.33
Anonymized_18	Hearing Loss (Unaided)	72.93
Anonymized_19	Hearing Loss (Unaided)	65.36
Anonymized_20	Hearing Loss (Unaided)	65.36
Anonymized_21	Hearing Loss (Unaided)	67.6
Anonymized_22	Hearing Loss (Unaided)	62.33
Anonymized_23	Hearing Loss (Unaided)	70.11
Anonymized_24	Hearing Loss (Unaided)	82.71
Anonymized_25	Hearing Loss (Unaided)	70.11
Anonymized_26	Hearing Loss (Unaided)	73.87

Anonymized_27	Hearing Loss (Unaided)	70.11
Anonymized_28	Hearing Loss (Unaided)	70.11
Anonymized_29	Hearing Loss (Unaided)	73.87
Anonymized_30	Hearing Loss (Unaided)	67.6
Anonymized_31	Hearing Loss (Unaided)	72.64

Supplementary Material 3

The figure displays the optode montage from Homer3; source optodes in red and detector optodes in blue. The table displays the MNI coordinates averaged across all participants and sessions for each channel (i.e., source-detector pair).

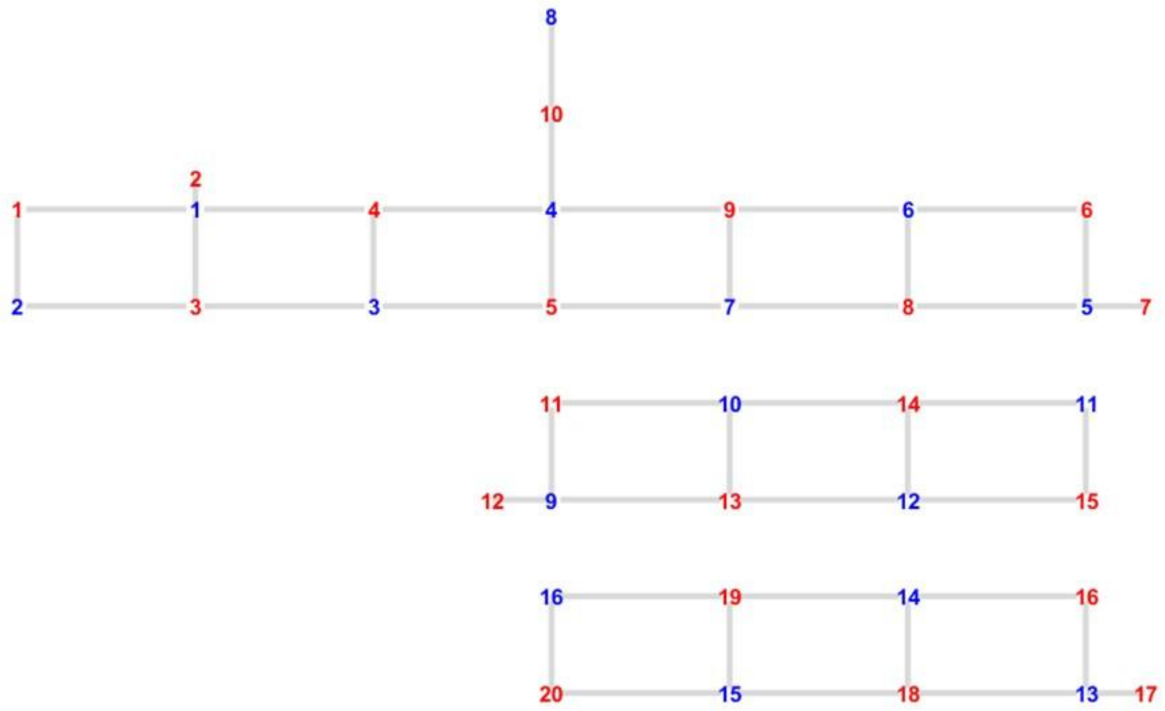

Channel	Source Optode	Detector Optode	MNI (X)	MNI (Y)	MNI (Z)
S1D1	1	1	44	35	28
S1D2	1	2	49	34	17
S2D1	2	1	32	41	33
S3D1	3	1	37	48	23
S3D2	3	2	45	45	10
S3D3	3	3	29	58	12
S4D1	4	1	24	50	29
S4D3	4	3	19	57	19
S4D4	4	4	12	57	29
S5D3	5	3	12	64	11
S5D4	5	4	3	58	19
S5D7	5	7	-7	61	10

S6D5	6	5	-38	36	15
S6D6	6	6	-38	33	26
S7D5	7	5	-38	41	6
S8D5	8	5	-38	47	9
S8D6	8	6	-31	44	21
S8D7	8	7	-23	56	10
S9D4	9	4	-6	56	28
S9D6	9	6	-19	49	28
S9D7	9	7	-12	55	16
S10D4	10	4	3	49	36
S10D8	10	8	2	34	55
S11D9	11	9	41	-42	57
S11D10	11	10	43	-49	54
S12D9	12	9	49	-29	56
S13D9	13	9	51	-28	49
S13D10	13	10	53	-35	46
S13D12	13	12	59	-20	33
S14D10	14	10	54	-45	38
S14D11	14	11	57	-40	18
S14D12	14	12	57	-31	24
S15D11	15	11	60	-26	3
S15D12	15	12	60	-16	12
S16D13	16	13	-52	-25	0
S16D14	16	14	-56	-17	9
S17D13	17	13	-56	-39	-1
S18D13	18	13	-52	-37	11
S18D14	18	14	-52	-28	19
S18D15	18	15	-51	-46	34
S19D14	19	14	-52	-21	30
S19D15	19	15	-50	-34	42
S19D16	19	16	-44	-25	42
S20D15	20	15	-38	-47	49
S20D16	20	16	-40	-37	56

Supplementary Material 4

Hearing Aid Style	Brand/Model
Behind the Ear (BTE)	OTICON MORE
Behind the Ear (BTE)	OPTICON OPN
Behind the Ear (BTE)	PHONAK
Behind the Ear (BTE)	SIGNIA
Behind the Ear (BTE)	UNICRON
Behind the Ear (BTE)	OTICON OPNS1
Behind the Ear (BTE)	KIRKLAND
Behind the Ear (BTE)	JABRA
In the Canal (ITC)	PHONAK VIRTO M50-312
In the Canal (ITC)	OTICON
In the Canal (ITC)	OTICON
In the Canal (ITC)	SIGNIA SILK 7XCIC DIGITAL
In the Canal (ITC)	OTICON, MINIRITE
In the Ear (ITE)	OTICON

Supplementary Material 5

Calm File Names	Happy File Names	Sad File Names	Angry File Names
03-01-02-01-01-01-02.wav	03-01-03-01-01-01-02.wav	03-01-04-01-01-01-02.wav	03-01-05-01-01-01-02.wav
03-01-02-01-01-01-08.wav	03-01-03-01-01-01-08.wav	03-01-04-01-01-01-08.wav	03-01-05-01-01-01-08.wav
03-01-02-01-01-01-14.wav	03-01-03-01-01-01-14.wav	03-01-04-01-01-01-14.wav	03-01-05-01-01-01-14.wav
03-01-02-01-01-01-18.wav	03-01-03-01-01-01-18.wav	03-01-04-01-01-01-18.wav	03-01-05-01-01-01-18.wav
03-01-02-01-01-01-20.wav	03-01-03-01-01-01-20.wav	03-01-04-01-01-01-20.wav	03-01-05-01-01-01-20.wav
03-01-02-01-01-02-02.wav	03-01-03-01-01-02-02.wav	03-01-04-01-01-02-02.wav	03-01-05-01-01-02-02.wav
03-01-02-01-01-02-08.wav	03-01-03-01-01-02-08.wav	03-01-04-01-01-02-08.wav	03-01-05-01-01-02-08.wav
03-01-02-01-01-02-14.wav	03-01-03-01-01-02-14.wav	03-01-04-01-01-02-14.wav	03-01-05-01-01-02-14.wav
03-01-02-01-01-02-18.wav	03-01-03-01-01-02-18.wav	03-01-04-01-01-02-18.wav	03-01-05-01-01-02-18.wav
03-01-02-01-01-02-20.wav	03-01-03-01-01-02-20.wav	03-01-04-01-01-02-20.wav	03-01-05-01-01-02-20.wav
03-01-02-01-02-01-02.wav	03-01-03-01-02-01-02.wav	03-01-04-01-02-01-02.wav	03-01-05-01-02-01-02.wav
03-01-02-01-02-01-08.wav	03-01-03-01-02-01-08.wav	03-01-04-01-02-01-08.wav	03-01-05-01-02-01-08.wav
03-01-02-01-02-01-14.wav	03-01-03-01-02-01-14.wav	03-01-04-01-02-01-14.wav	03-01-05-01-02-01-14.wav
03-01-02-01-02-01-18.wav	03-01-03-01-02-01-18.wav	03-01-04-01-02-01-18.wav	03-01-05-01-02-01-18.wav
03-01-02-01-02-01-20.wav	03-01-03-01-02-01-20.wav	03-01-04-01-02-01-20.wav	03-01-05-01-02-01-20.wav
03-01-02-01-02-02-02.wav	03-01-03-01-02-02-02.wav	03-01-04-01-02-02-02.wav	03-01-05-01-02-02-02.wav
03-01-02-01-02-02-08.wav	03-01-03-01-02-02-08.wav	03-01-04-01-02-02-08.wav	03-01-05-01-02-02-08.wav
03-01-02-01-02-02-14.wav	03-01-03-01-02-02-14.wav	03-01-04-01-02-02-14.wav	03-01-05-01-02-02-14.wav
03-01-02-01-02-02-18.wav	03-01-03-01-02-02-18.wav	03-01-04-01-02-02-18.wav	03-01-05-01-02-02-18.wav
03-01-02-01-02-02-20.wav	03-01-03-01-02-02-20.wav	03-01-04-01-02-02-20.wav	03-01-05-01-02-02-20.wav
03-01-02-02-01-01-02.wav	03-01-03-02-01-01-02.wav	03-01-04-02-01-01-02.wav	03-01-05-02-01-01-02.wav

03-01-02-02-01-01-08.wav	03-01-03-02-01-01-08.wav	03-01-04-02-01-01-08.wav	03-01-05-02-01-01-08.wav
03-01-02-02-01-01-14.wav	03-01-03-02-01-01-14.wav	03-01-04-02-01-01-14.wav	03-01-05-02-01-01-14.wav
03-01-02-02-01-01-18.wav	03-01-03-02-01-01-18.wav	03-01-04-02-01-01-18.wav	03-01-05-02-01-01-18.wav
03-01-02-02-01-01-20.wav	03-01-03-02-01-01-20.wav	03-01-04-02-01-01-20.wav	03-01-05-02-01-01-20.wav
03-01-02-02-01-02-02.wav	03-01-03-02-01-02-02.wav	03-01-04-02-01-02-02.wav	03-01-05-02-01-02-02.wav
03-01-02-02-01-02-08.wav	03-01-03-02-01-02-08.wav	03-01-04-02-01-02-08.wav	03-01-05-02-01-02-08.wav
03-01-02-02-01-02-14.wav	03-01-03-02-01-02-14.wav	03-01-04-02-01-02-14.wav	03-01-05-02-01-02-14.wav
03-01-02-02-01-02-18.wav	03-01-03-02-01-02-18.wav	03-01-04-02-01-02-18.wav	03-01-05-02-01-02-18.wav
03-01-02-02-01-02-20.wav	03-01-03-02-01-02-20.wav	03-01-04-02-01-02-20.wav	03-01-05-02-01-02-20.wav
03-01-02-02-02-01-02.wav	03-01-03-02-02-01-02.wav	03-01-04-02-02-01-02.wav	03-01-05-02-02-01-02.wav
03-01-02-02-02-01-08.wav	03-01-03-02-02-01-08.wav	03-01-04-02-02-01-08.wav	03-01-05-02-02-01-08.wav
03-01-02-02-02-01-14.wav	03-01-03-02-02-01-14.wav	03-01-04-02-02-01-14.wav	03-01-05-02-02-01-14.wav
03-01-02-02-02-01-18.wav	03-01-03-02-02-01-18.wav	03-01-04-02-02-01-18.wav	03-01-05-02-02-01-18.wav
03-01-02-02-02-01-20.wav	03-01-03-02-02-01-20.wav	03-01-04-02-02-01-20.wav	03-01-05-02-02-01-20.wav
03-01-02-02-02-02-02.wav	03-01-03-02-02-02-02.wav	03-01-04-02-02-02-02.wav	03-01-05-02-02-02-02.wav
03-01-02-02-02-02-08.wav	03-01-03-02-02-02-08.wav	03-01-04-02-02-02-08.wav	03-01-05-02-02-02-08.wav
03-01-02-02-02-02-14.wav	03-01-03-02-02-02-14.wav	03-01-04-02-02-02-14.wav	03-01-05-02-02-02-14.wav
03-01-02-02-02-02-18.wav	03-01-03-02-02-02-18.wav	03-01-04-02-02-02-18.wav	03-01-05-02-02-02-18.wav
03-01-02-02-02-02-20.wav	03-01-03-02-02-02-20.wav	03-01-04-02-02-02-20.wav	03-01-05-02-02-02-20.wav
